# Interactions with a Complex Microbiota Mediate a Trade-Off between the Host Development Rate and Heat Stress Resistance

**DOI:** 10.3390/microorganisms8111781

**Published:** 2020-11-13

**Authors:** Samuel Slowinski, Isabella Ramirez, Vivek Narayan, Medha Somayaji, Maya Para, Sarah Pi, Niharika Jadeja, Siavash Karimzadegan, Barbara Pees, Michael Shapira

**Affiliations:** 1 Department of Integrative Biology, 3040 Valley Life Sciences Building, University of California, Berkeley, CA 94720-3140, USA; isamasako@berkeley.edu (I.R.); viveknarayan@berkeley.edu (V.N.); msomayaji@berkeley.edu (M.S.); mpara@berkeley.edu (M.P.); piyage@berkeley.edu (S.P.); niharikajadeja@berkeley.edu (N.J.); sk2188@berkeley.edu (S.K.); bpees@berkeley.edu (B.P.); 2 Department of Biology, 4223 Biology-Psychology Bldg., University of Maryland, College Park, MD 20742, USA

**Keywords:** microbiome, microbiota, life-history traits, tradeoffs, *C. elegans*, development, heat stress

## Abstract

Animals and plants host diverse communities of microorganisms, and these microbiotas have been shown to influence host life history traits. Much has been said about the benefits that host-associated microbiotas bestow on the host. However, life history traits often demonstrate tradeoffs among one another. Raising *Caenorhabditis elegans* nematodes in compost microcosms emulating their natural environment, we examined how complex microbiotas affect host life history traits. We show that soil microbes usually increase the host development rate but decrease host resistance to heat stress, suggesting that interactions with complex microbiotas may mediate a tradeoff between host development and stress resistance. What element in these interactions is responsible for these effects is yet unknown, but experiments with live versus dead bacteria suggest that such effects may depend on bacterially provided signals.

## 1. Introduction

Animals and plants host and interact with diverse and often complex microbial communities, which can have substantial effects on the phenotype, fitness, and health of their hosts. In particular, microbes can profoundly influence key life history traits that determine host fitness, including enhanced resistance to pathogens [1,2,3,4], accelerated development [5,6,7], extended lifespan [8,9], and altered fecundity [9]. Because organisms have limited resources, tradeoffs are often observed between life history phenotypes [10,11,12,13]. Taken together, the observation that microbial symbionts often affect host life history traits and the observation that tradeoffs between life history traits are common raises the intriguing possibility that microbial symbionts may mediate host life history tradeoffs.

Different model systems offer distinct advantages in approaching fundamental questions in microbiome research. The nematode *Caenorhabditis elegans* offers the opportunity to work with self-replicating genetically homogenous populations, which averages out the inter-individual variation in microbiome composition. This has enabled the characterization of a conserved core gut microbiota, and enabled quantifying the respective contributions of environmental or genetic factors to its composition, identifying a significant contribution of host genetics [14,15,16,17,18]. In addition, its rampant reproduction and short generation time makes *C. elegans* useful in studies of the shaping of life history traits [19,20]. Lastly, the practice of bleaching gravid worms to obtain synchronized populations of germ-free larvae [21] makes the worm a simple gnotobiotic model and facilitates the manipulation of its microbiota. *C. elegans* is a bacterivore, hence the microbes in its environment serve both as a source of energy and nutrition as well as the seed for its gut microbiota. In laboratory studies, *C. elegans* is typically grown on a monoxenic culture of the *Escherichia coli* strain OP50, which is incapable of colonizing healthy worms [21]. However, in natural environments *C. elegans* was found to harbor numerous bacterial species, many of which have been isolated and have been shown to influence the host development rate, fecundity, and lifespan [22,23].

While individual bacterial isolates have been shown to influence *C. elegans* phenotypes, the net effects of a diverse naturalistic soil microbial community on *C. elegans* life history traits are less well studied. In the present study, we evaluated the effects of whole microbial communities on *C. elegans* life history traits by comparing the development rate and heat stress resistance of *C. elegans* raised either on complex compost microbial communities or on largely monoxenic cultures of *E. coli*. Because *E. coli* does not establish and persist as a commensal in the *C. elegans* gut, this experimental design allowed a comparison of host populations interacting with a complex microbial community with populations deprived of such interactions. Our results demonstrate a microbiota-driven tradeoff between accelerated development and compromised heat stress resistance. Furthermore, by comparing the development rate of *C. elegans* raised on live versus dead bacteria, we provide evidence that bacterially provided signals and not just nutrition mediate the effects of the microbial food source on the *C. elegans* development rate.

## 2. Materials and Methods

### 2.1. Strains Used

We used the wildtype *C. elegans* strain N2, previously obtained from the *Caenorhabditis* Genetics Center, *E. coli* strain OP50-1, a streptomycin-resistant derivative of OP50, and the *C. elegans* gut isolate *Enterobacter hormaecheli* strain *CEent1* (previously described as *E. cloacae* [14]).

### 2.2. Compost Microcosms

Microcosms were prepared as previously described [14,15] (Figure S1). Briefly, soil was collected from a *Eucalyptus* grove (37.8713 N, 122.2641 E) or from a redwood grove (37.8706 N, 122.263 E) on the University of California Berkeley campus. Soil was kept in plastic containers and kept aerated through cotton wool-plugged holes in their lids. Chopped organic produce (banana peel or banana fruit) was added to each soil container (~1:2 produce:soil by volume) and the mixture was left at room temperature to compost for two weeks, while being kept moist and stirred weekly. Soil samples were autoclaved prior to experiments to cure them of most of the native micro-organisms and all invertebrates and reconstituted either with a microbial extract from an unautoclaved sample of the same compost batch (1.5 mL supernatant from ~10 g soil in 40 mL M9) or with an excess of *E. coli* food (1.5 mL, saturated LB culture, 10× or 50× concentrated). Microcosm environments were incubated overnight at 25 °C to equilibrate prior to the addition of worms.

### 2.3. Growing Worms

Microcosms were seeded with 600–1000 germ-free eggs obtained by bleaching gravid worms or with L1 larvae obtained by bleaching gravid worms and allowing eggs to hatch and arrest on unseeded standard worm growth NGM plates. Worms were raised in microcosms at temperatures and for durations further designated.

### 2.4. Harvesting

Worms were harvested from soil using a Baermann funnel, as described elsewhere [15,24].

### 2.5. Development Rate

Worms harvested from soil were transferred to standard NGM plates and scored under the stereoscope for developmental stage. At least 20 worms were scored at each extraction time point for each treatment combination.

### 2.6. Heat Resistance Assays

Following harvesting from microcosms, L4 or young adult wildtype hermaphrodites, as determined morphologically, were singled to *E. coli*-seeded NGM plates, where they were heat shocked for 6 h at 31 °C (or maintained at 20 °C as controls) and then raised at 20 °C and transferred to fresh plates daily until they stopped laying eggs. Although soil microbial treatment affected the development rate, the worms were stage-matched by selecting only L4 or young adult worms for transfer to the heat shock assay at the start of the heat stress assay. The proportions of worms in the L4 and young adult stage at the start of the heat shock assays were balanced across treatments. The total offspring from each worm was counted as a measure of fecundity.

### 2.7. Development Rate Assays on Live Versus Dead Bacteria

Fifty germ-free wildtype L1 larvae were added to *E. coli-* or *CEent1*-seeded standard NGM plates. Bacteria were either alive, killed with the fixative agent paraformaldehyde (PFA), or killed with the antibiotic kanamycin, which inhibits protein synthesis in bacteria but is nontoxic to worms. In the PFA-killed treatments, stationary phase bacterial cultures were treated by incubation at 37 °C for 40 min in a 4% PFA solution and washed 5 times to remove any residual PFA. In the kanamycin-killed treatment, bacteria were grown to stationary phase in LB with no antibiotics, then diluted 5× in fresh LB + 400 µg/mL kanamycin and incubated overnight, followed by 50× concentration (to achieve a 10× concentration of the original culture) and seeding on 100 µg/mL kanamycin NGM plates. Bacteria killing was verified by observing no bacterial growth on LB plates.

### 2.8. Statistical Methods

Fecundity assays were statistically evaluated using a 2-way ANOVA (soil microbial treatment and heat treatment as factors), complemented with post-hoc independent sample *t*-tests. Development time series in native microbiota microcosms versus *E. coli* microcosms were compared with binomial logistic regressions. Comparisons of the single time point developmental stage compositions in worm populations raised in native-microbiota microcosms versus *E. coli* microcosms were evaluated using Chi-squared tests. The development rate on plates with live versus dead *E. coli* or *CEent1* was analyzed using a Kaplan–Meier analysis followed by log rank (Mantel–Cox) tests with post hoc pairwise comparisons. Similar analyses were carried out for the survival curves. All the statistical tests were carried out in IBM SPSS Statistics Version 25.

## 3. Results

### 3.1. Naturally Associated C. elegans Microbes Accelerate Worm Development

**Figure 1:**
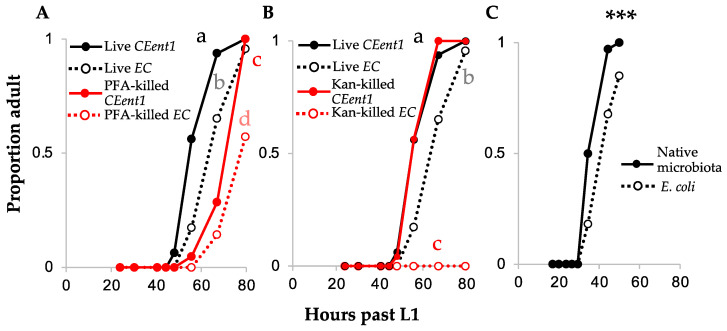
Naturally associated *C. elegans* microbes accelerate worm development dependent on the viability of the microbes. Shown are development time-courses (15 °C) for wildtype worms raised on *E. coli (EC)* or on the *Enterobacter hormaechei* worm gut isolate (*CEent1*), live, PFA-killed (**A**), or kanamycin-killed (**B**); shown are the results for one experiment, separated into two panels for clarity; *n* = 20 worms per group. Results shown in B were reproduced in 3 independent experiments (see Figure S2). Letters indicate statistically different curves (Kaplan–Meier analyses, post hoc *p*-values < 0.004). (**C**) Complex soil microbiotas similarly accelerate host development. A representative experiment (of three experiments with similar results (see Table S1), and of numerous unquantified observations) of wildtype worms raised in soil microcosms at 20 °C. *n* = 33–78 worms per group per time point. For the microcosm experiment (**C**), one replicate microcosm extracted per treatment per time point. ***, *p* < 0.001 between curves (binomial logistic regression).

Previous observations have indicated that bacteria naturally associated with *C. elegans*, including gut isolates such as the *Enterobacter hormaecheli* strain *CEent1* (previously described as *E. cloacae*), accelerated host development when provided as the sole source of food/interactions as compared to the non-colonizing *E. coli*. What component of these bacteria is responsible for promoting worm development is unknown. To examine whether this was due to favorable nutritional value or due to development-promoting bacterial signals, we compared the development of worms raised on live bacteria and those raised on bacteria killed either by paraformaldehyde fixation or by antibiotics treatment (kanamycin). We found that bacteria killed by chemical fixation were less capable of promoting development than live bacteria (Figure 1A). This was true both for growth on *E. coli* and growth on *CEent1*. However, dead *CEent1* maintained their relative advantage in promoting faster development compared to dead *E. coli.* Killing bacteria with kanamycin resulted in more extreme differences in developmental effects. Kanamycin-killed *E. coli* could not support worm development, with none or only a few worms reaching adulthood at the examined time (although kanamycin-killed *E. coli* supports adult survival [25]); kanamycin-killed *CEent1*, on the other hand, maintained the same ability to promote development as live *CEent1* (Figure 1B and Figure S2). Together, these results suggest that nutritional value alone is not sufficient to support worm development (kanamycin-killed *E. coli*) and that worm development additionally requires bacterially provided signals. It seems that those differ between different bacteria, as does their preservation under different chemical treatments—i.e., *CEent1*-provided signals resist kanamycin treatment, but not fixation, and *E. coli*-provided signals are damaged to different extents by both treatments.

Given the varying effects of bacterial signals provided by different bacteria on worm development, we wished to examine how such signals combined in natural-like environments with complex microbial communities affected worm development. To this end, worms were raised in compost microcosms emulating the worm natural environment, in which autoclaved compost was reconstituted with the original native microbiota or with *E. coli* alone [15]. Under these conditions, interactions with complex microbiomes resulted in faster development in most experiments (Figure 1C, Table S1), but there are exceptions (see below). Given that the food availability was similar for both treatments (as indicated by lack of larval arrest in any of the treatments, see also Figure S2) our interpretation of these results is that interactions of *C. elegans* with its naturally associated bacteria contribute to a faster development than its better-characterized development in the presence of *E. coli*. This contribution appears to be non-additive, since the rate of development in worms raised on complex microbiotas was comparable to that in worms raised on *CEent1* alone (Figure 1C compared to Figure S2).

### 3.2. Microbiota-Enhanced Developmental Rate Demonstrates a Trade-Off with Heat Stress Resistance

**Figure 2:**
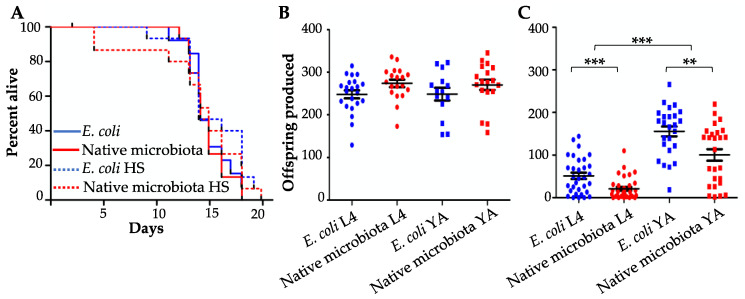
Growth on a complex microbiota reduces fecundity following heat stress. (**A**) Lifespan of worms with or without heat stress (HS). Data pooled across 2 experiments (20 °C, *n* = 13–15 worms per group per experiment; NS, Log rank test). (**B**,**C**) Fecundity of worms raised in compost microcosms, transferred to plates as L4 larvae or young adults (YA) and maintained at 20 °C (**B**), or heat-shocked at 31 °C for 6 h at the designated stage (**C**). Each dot represents the lifetime offspring produced by a single worm. Error bars represent ± standard error of the mean. Data pooled across 5 replicate experiments. ** *p* < 0.01, *** *p* < 0.001, *t*-test.

The faster development on naturally associated bacteria and microbiotas represents a fitness advantage for the worm. However, fitness advantages manifested in early life traits often trade off with performance in late-life traits, representing the conflict between investment in germline versus soma [26]. We examined whether this might be the case also with the enhanced developmental rate observed in worms interacting with a complex microbiota. We focused on worm resistance to mild heat stress (31 °C, 6 h). Thermotolerance has previously been found to predict longevity [27,28,29]. Interactions with complex microbiotas during development did not significantly affect subsequent worm lifespan on *E. coli* plates, with or without post-developmental heat stress (Figure 2A). Complex microbiotas also did not significantly affect fecundity under benign temperature conditions (Figure 2B). However, development on complex natural-like microbiotas significantly reduced fecundity of worms following heat stress (Figure 2C). This held true regardless of the exact developmental stage in which heat stress was initiated, which turned out to be more disruptive to reproduction when initiated at the L4 larval stage, similar to previous observations [30]. This suggests that faster development on complex microbiotas was associated with reduced fecundity, but only when heat stress was further applied, implying a microbiota-dependent trade-off between fast development and heat stress resistance.

**Figure 3:**
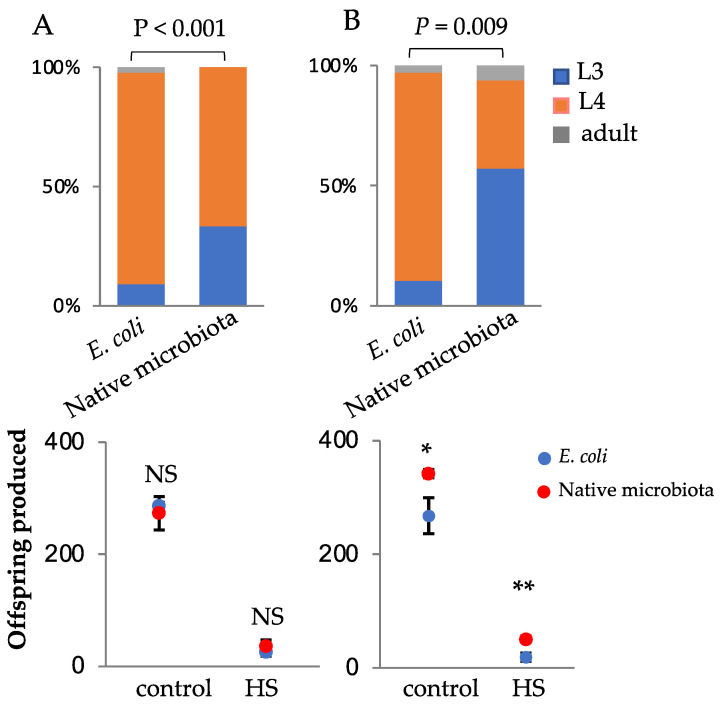
Support for an inverse relationship between accelerated development and heat stress resistance. Shown are two instances (**A**,**B**) where worms raised on complex microbiotas showed slower development than those raised in *E. coli*-only microcosms. Top, developmental stages after 43 h (20 °C); Fisher exact test. Bottom, fecundity with or without heat stress (HS, 31 °C for 6 h). Error bars represent ± standard error of the mean. * *p* < 0.05, ** *p* < 0.01, *t*-tests. Fecundity data was obtained from worms exposed to heat stress at the L4 stage. *n* = 20 worms per treatment in each soil type.

Exceptions to the rule can often provide support for the rule. While the vast majority of microcosm experiments showed a faster development of worms raised on complex microbiotas, in two separate experiments worms raised on microcosms with a complex microbiota actually showed slower development than those raised on *E. coli*-only microcosms (Figure 3). In one of these experiments, the fecundity did not differ regardless of the soil microbial treatment, with or without heat stress (Figure 3A). In the second, worms raised on complex microbiota showed increased fecundity compared to those raised on *E. coli*, regardless of heat treatment (Figure 3B). In both cases, there was no significant interaction between heat stress and soil microbial treatment, indicating that soil microbial communities that do not increase the development rate also do not affect the heat stress resistance.

## 4. Discussion

Studies of the interactions between animals and their associated microbial communities often focus on microbe-specific benefits. Detrimental effects on health or fitness are typically considered as a consequence of imbalances in microbiome composition or dysbiosis. However, our results imply that benefits manifested in one life history trait, attributed to interactions with microbes in an animal’s natural environment, can trade off with another life history trait. Specifically, the accelerated development in *C. elegans* promoted by interactions with a natural-like and complex soil community shows a trade-off with fecundity following heat stress. This is not simply a trade-off with fecundity, as the latter is not affected without additional stress, and therefore should be considered as a trade off with adult stress resistance. Thus, our results support a trade-off between an early life history trait (development) and a late-life history trait (adult stress resistance). While the soil microbiota usually accelerated development and reduced heat stress resistance, in two experiments interactions with a complex soil community demonstrated a reduction in the host development rate rather than acceleration. Setting up of these experiments was identical to those in which exposure to complex microbiotas accelerated development, but the soil was collected in different seasons, raising the possibility that the composition of those complex microbiotas might have been different. While the outlier experiments demonstrate that native microbiotas do not always accelerate development and reduce resistance to heat stress, they provide further support for the trade-off between development rate and stress resistance, as in these two experiments stress resistance was not affected. This demonstrates that the native microbiota reduces heat stress resistance, but only when it accelerates development.

Previous studies have described beneficial effects of microbes other than its *E. coli* food strain on *C. elegans* stress resistance. For example, Lee et al. [31] demonstrated that, when exposed to the toxic heavy metal cadmium, worms raised on a culturable subset of a soil microbial community had higher reproductive rates than worms raised on *E. coli*. In addition, biofilm-producing *Bacillus subtilis*, *Lactobacillus rhamnosus*, or *Pseudomonas fluorescens* increased *C. elegans* survival under heat stress [32]. In plants, the increased abundance of certain commensal bacteria was shown to be associated with enhanced stress resistance [33]. Our results provide a counter example to these patterns of microbiome-enhanced stress resistance, showing that sometimes a diverse and naturalistic microbiota can reduce stress resistance.

How exactly bacteria affect worm development is still not known. However, development on dead commensals or food bacteria suggest that bacterially provided signals (i.e., microbe-associated molecular patterns), in addition to the calories and nutrients provided by the microbes, take part in mediating these effects. Previous research has suggested that bacterial signaling molecules may play an important role in regulating the *C. elegans* development rate. For example, it has been previously shown that *C. elegans* grown on axenic culture exhibit delayed development, smaller body size, and reduced fecundity [34,35,36]. Furthermore, MacNeil, Watson, Arda, Zhu, and Walhout [22] demonstrated that even a very small amount of live *Comamonas* bacteria added to a lawn of *E. coli* could induce an increased development rate, suggesting that *Comamonas* had a dominant effect over *E. coli* on development rate and that the differences in the development rate of *Comamonas* versus *E. coli* do not simply reflect differences in caloric intake. How the worm recognizes the bacterial patterns is not known. *C. elegans* lacks homologs for most known pattern recognition receptors, excluding several proteins involved in viral recognition [37,38] and one involved in sensing epidermal wounding and fungal infection [39]. However, circumstantial evidence previously suggested that the recognition of microbe-associated molecular patterns (MAMPs) does occur, potentially aiding worms’ ability to distinguish between non-pathogenic bacteria and pathogens [25]. The requirement that we identified here for signals beyond nutrients to promote host development provides new support for MAMP recognition in *C. elegans* and suggests that such recognition is required not only for immune responses but also for interactions with commensals.

Our results build on previous work in other systems that offered evidence of microbe-dependent tradeoffs between host life history traits. For example, Emelianoff et al. [40] found that bacterial *Xenorhabdus* symbionts reduced the survival of their entomopathogenic *Steinernema* nematode host but increased host reproductive success. Similarly, Walters et al. [41] demonstrated that isogenic lines of *Drosophila melanogaster* reared with different bacteria varied their investment in early reproduction versus somatic maintenance. How common it is for microbial symbionts to mediate life history tradeoffs in their hosts and to alter the relative investment in early and late-life traits and the mechanisms by which they do so are interesting topics for future research.

## Supplementary Materials

The following are available online at https://www.mdpi.com/2076-2607/8/11/1781/s1: Table S1: Complex soil microbiotas accelerate host development. Figure S1: Experimental microcosms in 20 mL glass vials. Figure S2: Kanamycin treatment of *E. coli* and *Enterobacter hormaecheli CEent1* differentially affects *C. elegans* development.

## References

[1] Montalvo-Katz S., Huang H., Appel M.D., Berg M., Shapira M. (2013). Association with soil bacteria enhances p38-dependent infection resistance in *Caenorhabditis elegans*. Infect. Immun..

[2] Endt K., Stecher B., Chaffron S., Slack E., Tchitchek N., Benecke A., Van Maele L., Sirard J.C., Mueller A.J., Heikenwalder M. (2010). The microbiota mediates pathogen clearance from the gut lumen after non-typhoidal salmonella diarrhea. PLoS Pathog..

[3] Stecher B., Chaffron S., Kappeli R., Hapfelmeier S., Freedrich S., Weber T.C., Kirundi J., Suar M., McCoy K.D., Von Mering C. (2010). Like will to like: Abundances of closely related species can predict susceptibility to intestinal colonization by pathogenic and commensal bacteria. PLoS Pathog..

[4] Thomason C.A., Mullen N., Belden L.K., May M., Hawley D.M. (2017). Resident microbiome disruption with antibiotics enhances virulence of a colonizing pathogen. Sci. Rep..

[5] Bakula M. (1969). The persistence of a microbial flora during postembryogenesis of *Drosophila melanogaster*. J. Invertebr. Pathol..

[6] Shin S.C., Kim S.H., You H., Kim B., Kim A.C., Lee K.A., Yoon J.H., Ryu J.H., Lee W.J. (2011). *Drosophila* microbiome modulates host developmental and metabolic homeostasis via insulin signaling. Science.

[7] Dirksen P., Assié A., Zimmermann J., Zhang F., Tietje A.-M., Marsh S.A., Félix M.-A., Shapira M., Kaleta C., Schulenburg H. (2020). CeMbio-The Caenorhabditis elegans microbiome resource. G3 Genes Genomes Genet..

[8] Han B., Sivaramakrishnan P., Lin C.C.J., Neve I.A.A., He J.Q., Tay L.W.R., Sowa J.N., Sizovs A., Du G.W., Wang J. (2017). Microbial genetic composition tunes host longevity. Cell.

[9] Rosengaus R.B., Zecher C.N., Schultheis K.F., Brucker R.M., Bordenstein S.R. (2011). Disruption of the termite gut microbiota and its prolonged consequences for fitness. Appl. Environ. Microbiol..

[10] Stearns S.C. (1989). Trade-offs in life-history evolution. Funct. Ecol..

[11] Bonsall M.B., Mangel M. (2004). Life-history trade-offs and ecological dynamics in the evolution of longevity. Proc. R. Soc. B Biol. Sci..

[12] Hekimi S. (2006). How genetic analysis tests theories of animal aging. Nat. Genet..

[13] Partridge L., Gems D. (2006). Beyond the evolutionary theory of ageing, from functional genomics to evo-gero. Trends Ecol. Evol..

[14] Berg M., Zhou X.Y., Shapira M. (2016). Host-specific functional significance of *Caenorhabditis* gut commensals. Front. Microbiol..

[15] Berg M., Stenuit B., Ho J., Wang A., Parke C., Knight M., Alvarez-Cohen L., Shapira M. (2016). Assembly of the *Caenorhabditis elegans* gut microbiota from diverse soil microbial environments. ISME J..

[16] Zhang F., Berg M., Dierking K., Felix M.A., Shapira M., Samuel B., Schulenburg H. (2017). *Caenorhabditis elegans* as a model for microbiome research. Front. Microbiol..

[17] Dirksen P., Marsh S.A., Braker I., Heitland N., Wagner S., Nakad R., Mader S., Petersen C., Kowallik V., Rosenstiel P. (2016). The native microbiome of the nematode *Caenorhabditis elegans*: Gateway to a new host-microbiome model. BMC Biol..

[18] Berg M., Monnin D., Cho J., Nelson L., Crits-Christoph A., Shapira M. (2019). TGFβ/BMP immune signaling affects abundance and function of *C. elegans* gut commensals. Nat. Commun..

[19] Gray J.C., Cutter A.D. (2014). Mainstreaming *Caenorhabditis elegans* in experimental evolution. Proc. R. Soc. B Biol. Sci..

[20] Zhang J.Y., Holdori A.D., Walhout A.J.M. (2017). *C. elegans* and its bacterial diet as a model for systems-level understanding of host-microbiota interactions. Curr. Opin. Biotechnol..

[21] Stiernagle T. (2006). Maintenance of *C. elegans*. WormBook.

[22] MacNeil L.T., Watson E., Arda H.E., Zhu L.J., Walhout A.J.M. (2013). Diet-Induced developmental acceleration independent of TOR and insulin in *C. elegans*. Cell.

[23] Samuel B.S., Rowedder H., Braendle C., Felix M.A., Ruvkun G. (2016). *Caenorhabditis elegans* responses to bacteria from its natural habitats. Proc. Natl. Acad. Sci. USA.

[24] Barriere A., Felix M.A. (2014). Isolation of *C. elegans* and related nematodes. WormBook.

[25] Twumasi-Boateng K., Shapira M. (2012). Dissociation of immune responses from pathogen colonization supports pattern recognition in *C. elegans*. PLoS ONE.

[26] Lemaitre J.F., Berger V., Bonenfant C., Douhard M., Gamelon M., Plard F., Gaillard J.M. (2015). Early-late life trade-offs and the evolution of ageing in the wild. Proc. R. Soc. B Biol. Sci..

[27] Lithgow G.J., White T.M., Melov S., Johnson T.E. (1995). Thermotolerance and extended life-span conferred by single-gene mutations and induced by thermal stress. Proc. Natl. Acad. Sci. USA.

[28] Johnson T.E., Cypser J., De Castro E., De Castro S., Henderson S., Murakami S., Rikke B., Tedesco P., Link C. (2000). Gerontogenes mediate health and longevity in nematodes through increasing resistance to environmental toxins and stressors. Exp. Gerontol..

[29] Labbadia J., Morimoto R.I. (2015). Repression of the heat shock response is a programmed event at the onset of reproduction. Mol. Cell.

[30] Zevian S.C., Yanowitz J.L. (2014). Methodological considerations for heat shock of the nematode *Caenorhabditis elegans*. Methods.

[31] Lee S., Kim Y., Choi J. (2020). Effect of soil microbial feeding on gut microbiome and cadmium toxicity in *Caenorhabditis elegans*. Ecotoxicol. Environ. Saf..

[32] Smolentseva O., Gusarov I., Gautier L., Shamovsky I., DeFrancesco A.S., Losick R., Nudler E. (2017). Mechanism of biofilm-mediated stress resistance and lifespan extension in *C. elegans*. Sci. Rep..

[33] Liu H.W., Brettell L.E., Qiu Z.G., Singh B.K. (2020). Microbiome-mediated stress resistance in plants. Trends Plant Sci..

[34] Vanfleteren J.R., Braeckman B.P. (1999). Mechanisms of life span determination in *Caenorhabditis elegans*. Neurobiol. Aging.

[35] Houthoofd K., Braeckman B.P., Johnson T.E., Vanfleteren J.R. (2003). Life extension via dietary restriction is independent of the Ins/IGF-1 signalling pathway in *Caenorhabditis elegans*. Exp. Gerontol..

[36] Houthoofd K., Braeckman B.P., Lenaerts I., Brys K., De Vreese A., Van Eygen S., Vanfleteren J.R. (2002). Axenic growth up-regulates mass-specific metabolic rate, stress resistance, and extends life span in *Caenorhabditis elegans*. Exp. Gerontol..

[37] Ashe A., Belicard T., Le Pen J., Sarkies P., Frezal L., Lehrbach N.J., Felix M.A., Miska E.A. (2013). A deletion polymorphism in the *Caenorhabditis elegans* RIG-I homolog disables viral RNA dicing and antiviral immunity. Elife.

[38] Coffman S.R., Lu J., Guo X., Zhong J., Jiang H., Broitman-Maduro G., Li W.-X., Lu R., Maduro M., Ding S.-W. (2017). *Caenorhabditis elegans* RIG-I homolog mediates antiviral RNA interference downstream of dicer-dependent biogenesis of viral small interfering RNAs. mBio.

[39] Zugasti O., Bose N., Squiban B., Belougne J., Kurz C.L., Schroeder F.C., Pujol N., Ewbank J.J. (2014). Activation of a G protein-coupled receptor by its endogenous ligand triggers the innate immune response of *Caenorhabditis elegans*. Nat. Immunol..

[40] Emelianoff V., Chapuis E., Le Brun N., Chiral M., Moulia C., Ferdy J.B. (2008). A survival-reproduction trade-off in entomopathogenic nematodes mediated by their bacterial symbionts. Evol. Int. J. Org. Evol..

[41] Walters A.W., Hughes R.C., Call T.B., Walker C.J., Wilcox H., Petersen S.C., Rudman S.M., Newell P.D., Douglas A.E., Schmidt P.S. (2020). The microbiota influences the *Drosophila melanogaster* life history strategy. Mol. Ecol..

