# Supplementary material for: Interactions with a Complex Microbiota Mediate a Trade-Off between the Host Development Rate and Heat Stress Resistance
Source: Microorganisms. 2020 Nov 13;8(11):1781. doi: 10.3390/microorganisms8111781 (PMC7697855; doi:10.3390/microorganisms8111781)
Supplement: Supplementary file 1 [file microorganisms-08-01781-s001.pdf]

Table S1. Complex soil microbiotas accelerate host development.

Experiment	Time to 50% adults (hrs) *E. coli*[a]	Time to 50% adults (hrs) native microbiota[b]	Difference (hrs)[d]	[c]*P*-value
1	40.8	34.4	6.4	<0.001
2	39.8	38.5	1.3	0.028
3	43.8	37.4	6.4	0.003

[a,b] Worms were raised in compost microcosms, or on autoclaved microcosms supplemented with *E. coli* alone (20°C).

[c] P-values designate statistical significance between development curves (binomial logistic regression analyses).

[d] On average, worm populations reached 50% adult 4.8 hours earlier when raised on complex microbiotas. Experiment 1 data previously shown (Figure 1C)

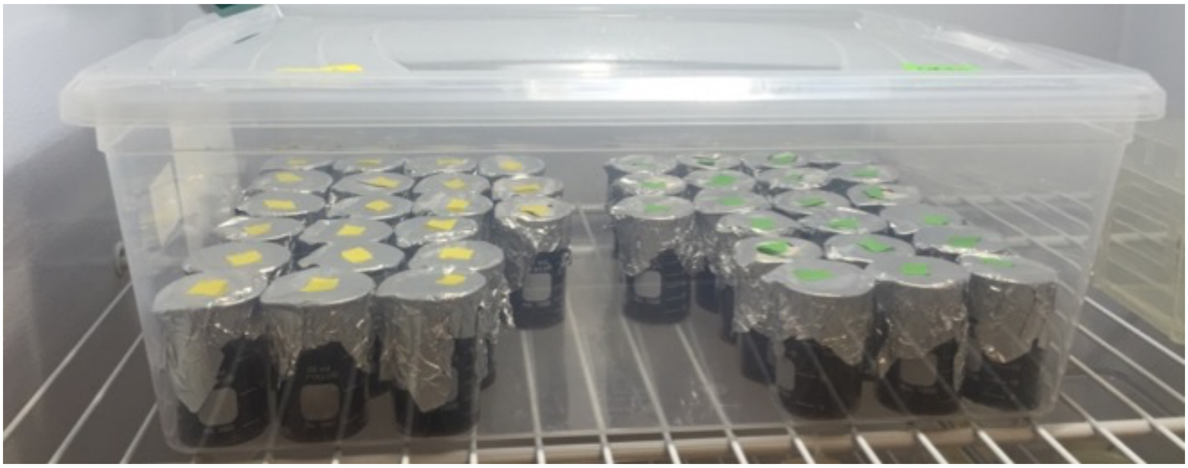

Figure S1. Experimental microcosms in 20 mL glass vials.

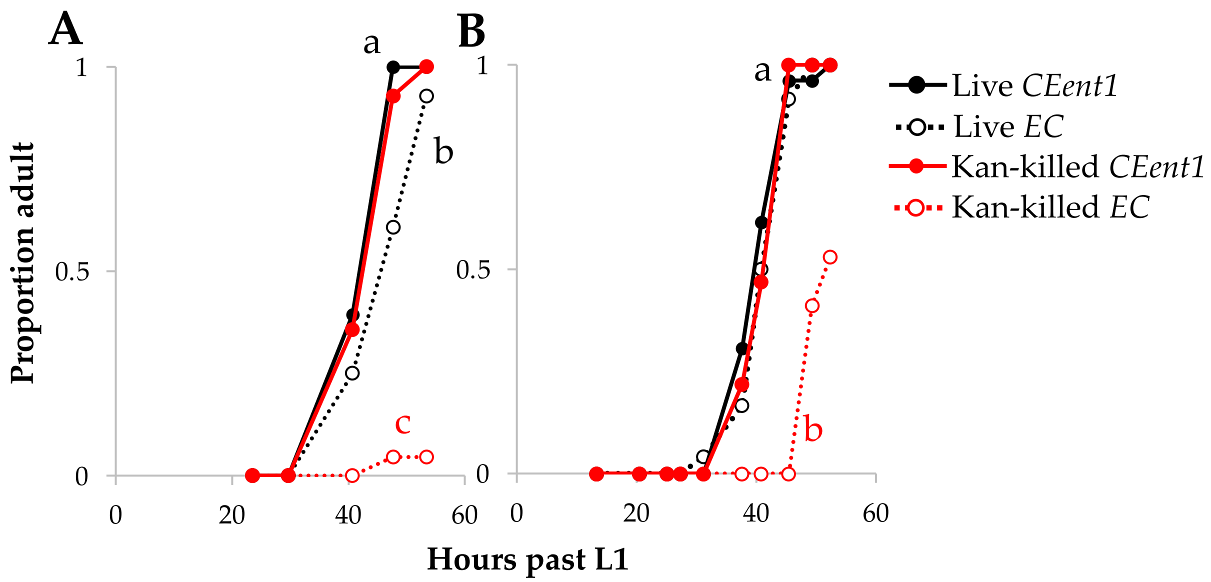

Figure S2. Kanamycin treatment of *E. coli* and *Enterobacter hormaecheli CEent1* differentially affects *C. elegans* development. Development rate of wildtype worms raised on designated bacterial strains in two additional experiments (A, B) to the one presented in Fig. 1 (20 °C). Letters adjacent to curves indicate statistically distinct classes (Kaplan-Meier analyses, post-hoc p-value < 0.002).
